# A Novel *Weizmannia coagulans* Strain WC412 with Superior Environmental Resilience Improves Growth Performance of Mice by Regulating the Intestinal Microbiota

**DOI:** 10.3390/ani15162446

**Published:** 2025-08-20

**Authors:** Xue Xiao, Hao Huang, Wendi Yu, Jun Liu, Yuanliang Hu, Xiang Yu, Xicai Zhang

**Affiliations:** 1 Hubei Key Laboratory of Edible Wild Plants Conservation and Utilization, College of Life Science, Hubei Normal University, Huangshi 435002, China; xuexiao1107@163.com (X.X.);; 2 National Key Laboratory of Agricultural Microbiology, College of Life Science and Technology, Huazhong Agricultural University, Wuhan 430070, China; hjhuanghao@163.com; 3 School of Biological Engineering, Jingchu University of Technology, Jingmen 448000, China

**Keywords:** *Weizmannia coagulans*, intestinal microbiota, strain selection, growth performance, immune modulation

## Abstract

The growing demand for sustainable and antibiotic-free animal production has intensified interest in probiotics as functional feed additives. In this study, novel strains of *Weizmannia coagulans* (WC412 and WC413) were isolated from pickle water—a previously unexplored source for probiotic screening. These isolates, along with three reference strains (*W. coagulans* S8, S15, and S17), were evaluated for their tolerance to heat, acid, and bile salts. Strain WC412 exhibited superior environmental resilience, as validated by principal component analysis (PCA) for comprehensive stress-tolerance assessment, and was selected for further investigations. A murine model was employed to assess the physiological and microbiological impacts of WC412 supplementation at varying doses. Medium-dose (1 × 10^7^ CFU·mL^−1^) administration significantly improved body weight gain by 13% (*p* < 0.05), modulated serum lipid profiles, and increased antioxidant enzyme activity and IgG/IL-2 levels (*p* < 0.05). Notably, WC412 uniquely enriched beneficial genera (e.g., *Fructilactobacillus* and *Limosilactobacillus*) and promoted metabolic pathways linked to short-chain fatty acid production, as revealed by 16S rDNA sequencing and Kyoto Encyclopedia of Genes and Genomes (KEGG) analysis. These findings highlight WC412 as a robust probiotic candidate for enhancing animal growth performance and gut health through novel microbiota-mediated mechanisms.

## Simple Summary

The overuse of antibiotics in animal farming has raised serious concerns about drug resistance and food safety. As a safer alternative, probiotics are gaining attention for their ability to promote animal health and growth. In this study, we isolated a beneficial bacterial strain, *Weizmannia coagulans* WC412, from fermented kimchi brine and tested its potential as a probiotic feed additive. WC412 could survive harsh conditions, like high heat, strong stomach acid, and bile salts, making it suitable for use in animal feeds. When fed to mice, WC412 improved body weight gain, boosted immune function, and promoted a healthier gut environment by increasing beneficial gut bacteria. These results suggest that WC412 is a promising candidate to support animal health and growth, offering a potential alternative to antibiotics in livestock and poultry farming.

## 1. Introduction

The global trend toward agricultural intensification has accelerated the adoption of intensive farming systems; however, high-density rearing environments markedly elevate the risk of disease transmission among livestock and poultry [1]. The application of antibiotics has substantially enhanced the control of infectious diseases within animal husbandry. Nonetheless, the widespread and unregulated use of antibiotics has precipitated multiple public health crises, most notably the pervasive emergence of antibiotic resistance [2]. Moreover, in the context of animal production, antibiotic administration should be strictly confined to therapeutic purposes and must not be employed as a technical expedient to increase stocking density, enhance animal conditions, or accelerate growth [3]. Owing to their multifaceted regulatory effects on the gastrointestinal tract, probiotics have emerged as a pivotal strategy to replace antibiotics in both poultry and aquaculture. Their growth-promoting properties and capacity to improve feed conversion efficiency have garnered considerable attention [4]. For instance, Muhammad et al. [5] reported that supplementation with a composite probiotic preparation of *Lactobacillus plantarum* and *Pediococcus pentosaceus* effectively enhanced the growth performance and optimized morphometric traits of *Oreochromis niloticus*. Similarly, Slizewska et al. [6] applied *lactic acid bacteria* (LAB) in broiler production and found that LAB supplementation modulated the intestinal microbiota structure, thereby significantly improving broiler production performance. Therefore, the development of probiotics as a novel alternative to antibiotics has become a major focus in the research and development of functional feed additives.

Commonly employed probiotics in animal husbandry encompass members of the genera *Lactobacillus*, *Bifidobacterium,* and *Bacillus subtilis*. While preparations based on *Lactobacillus* and *Bifidobacterium* predominate the market, many of these strains suffer from limited tolerance to extreme temperatures and heightened sensitivity to gastric acid and digestive enzymes [7,8]. In contrast, species within the genus *Bacillus* are intrinsically more resistant to adverse conditions, rendering them especially attractive as next-generation probiotic candidates. Among these, *Bacillus coagulans*—a Gram-positive, facultatively anaerobic, spore-forming bacterium capable of lactic acid synthesis—has earned the epithet “king of probiotics” [9]. Its spores withstand gastric acidity and, upon reaching the hypoxic environment of the intestine, germinate and proliferate. There, *B. coagulans* secretes the broad-spectrum antimicrobial peptide coagulin, which suppresses pathogenic gut bacteria [4]. Thanks to these characteristics, *B. coagulans* has been widely applied in both terrestrial and aquatic animal production systems, where it has been demonstrated to enhance growth performance, bolster immune responses, improve digestive function, and increase disease resistance [9,10,11]. Given these benefits, current research efforts are focused on isolating and characterizing *B. coagulans* strains with even greater stress resilience in order to expand the repertoire of probiotic resources and develop more robust functional feed additives.

The present study aimed to isolate and characterize bacterial strains from fermented kimchi brine. Morphological assessment and 16S rRNA gene sequencing confirmed that the isolates belonged to *W. coagulans*. The newly isolated strains were evaluated in parallel with three reference *W. coagulans* strains (S8, S15, and S17) preserved in our laboratory, and stress-resistance assays were performed to identify variants with enhanced environmental tolerance. Furthermore, a murine model was used to assess the impact of the candidate *W. coagulans* strains on host growth performance and modulation of the intestinal microbiota. By uncovering novel *W. coagulans* strains with enhanced stress resilience, this work not only expands the repository of probiotic candidates but also lays the theoretical groundwork for their potential applications in livestock, poultry, and aquaculture systems.

## 2. Materials and Methods

### 2.1. Materials

Pickle water was provided free of charge by Hongjin Agricultural and By-products Wholesale Market, Huangshi Harbor District (Huangshi, China). *W. coagulans* S5, S8, and S17 were conserved strains from the Laboratory of Microbial Resources Utilization and Processing, Hubei Normal University (Huangshi, China).

### 2.2. Ethics Statement

The animals used in the experiments were SPF-grade KM male mice purchased from Hubei Provincial Laboratory Animal Research Center (Wuhan, China) (No. 42000600057346). This study was approved by the Ethics Committee for Laboratory Animals and Welfare of Ningxia University (Yinchuan, China) (No. 20240017). All animal feeding and execution procedures were performed in accordance with the Chinese Guide for the Care and Use of Laboratory Animals.

### 2.3. Isolation and Identification of W. coagulans from Pickle Water

#### 2.3.1. Isolation of Strains

Following heat treatment of the kimchi brine at 80 °C, the sample was serially diluted and spread onto 1.5% (*w*/*v*) Nutrient Agar plates, composed of 5.0 g/L peptone, 3.0 g/L beef extract, and 15 g/L agar, supplemented with 0.006% bromocresol purple as a pH indicator. After incubation and colony development, presumptive screening was performed based on the presence or absence of hydrolytic halos surrounding the colonies, as well as differences in colony morphology. Briefly, 1 mL of pickle water was inoculated into 50 mL of enrichment medium (Bacillus megatherium medium, RuiChu Biotechnology Co., Ltd., Shanghai, China) and incubated at 45 °C with shaking at 180 r/min for 24 h. Then, 10 mL of the culture was treated in a water bath at 80 °C for 10 min, followed by serial dilution and plating onto bromocresol purple agar plates, and incubated at 45 °C for 24 h. Single colonies were picked and streaked onto the yeast extract peptone dextrose medium (YPD) solid medium, incubated at 45 °C for 24 h, and purified by streaking three times. The purified strains were stored in 30% glycerol at −80 °C [12].

#### 2.3.2. Morphological Identification

According to the method described by Madushanka et al. [13], the preserved strains were inoculated onto solid YPD medium and incubated at 45 °C for 24 h. Gram staining was performed using the Gram staining method, and the stained cells were observed under an oil immersion microscope (Olympus Corporation, Tokyo, Japan).

#### 2.3.3. Strain Identification

Genomic DNA of the bacterial isolates was extracted using a commercial DNA extraction kit (Sangon Biotech Co., Ltd., Shanghai, China). The 16S rDNA gene was targeted for amplification using universal primers 27F (5′-AGAGTTTGATCCTGGCTCAG-3′) and 1492R (5′-GGTTACCTTGTTACGACTT-3′). PCR amplification was performed under the following conditions: initial denaturation at 95 °C for 5 min, followed by 30 cycles of denaturation at 95 °C for 45 s, annealing at 52 °C for 45 s, and extension at 72 °C for 90 s. PCR products were separated by agarose gel electrophoresis, and the DNA bands were submitted to Aoke Dingsheng Biotechnology Co., Ltd. (Wuhan, China) for sequencing. The resulting sequences were aligned and compared using BLAST (2.16.0 National Center for Biotechnology Information, Bethesda, MD, USA) in the NCBI database (http://www.ncbi.nlm.nih.gov/blast/, accessed on 1 November 2024), and the strain was identified based on the sequence with the highest homology [14].

### 2.4. Evaluation of Bacterial Tolerance

#### 2.4.1. High Temperature Tolerance

Bacterial suspensions of the test strains were subjected to water bath treatments at 37 °C, 80 °C, 90 °C, and 100 °C for 10 min and 20 min, with the 37 °C group serving as the control. Thermal tolerance was assessed by calculating survival rates based on colony counts obtained through plate spreading [15].

#### 2.4.2. Acid Tolerance Measurement

Bacterial suspensions of the test strains were inoculated into PBS buffers with pH values of 2.0, 2.5, 3.0, and 7.0 at a ratio of 1:10, followed by incubation at 37 °C for 3 h. The pH 7.0 group served as the control. Acid tolerance was evaluated by determining survival rates through plate counting [16].

#### 2.4.3. Bile Salt Tolerance Assay

Bacterial suspensions of the test strains were added at a ratio of 1 to 10 to phosphate-buffered saline (PBS) buffers containing 0%, 0.1%, 0.3%, and 0.5% bile salts. The mixtures were incubated at 37 °C for 3 h. The group without bile salt served as the control. Survival rates were determined by plate counting to evaluate bile salt tolerance [16].

### 2.5. Mouse Feeding Model of W. coagulans

#### 2.5.1. Experimental Animals and Group Design

Forty KM mice were housed in an artificial climate-controlled room maintained at 25 ± 1 °C with a relative humidity of 45–50% under a 12 h light/dark cycle. Animals had ad libitum access to food and water, and bedding was replaced twice weekly to ensure hygienic conditions. After a 5-day acclimatization period, mice were randomly assigned to treatment groups using a stratified completely randomized design to ensure balanced baseline body weight across groups. The study was conducted as a completely randomized design (CRD), with treatment group as the main fixed effect and individual mouse as the experimental unit. No blocking factors were applied, and random effects were not incorporated due to the homogenous housing environment and the absence of clustering. Each mouse received a daily oral gavage of 0.1 mL of either sterile saline (control) or *W. coagulans* bacterial suspension. The experimental groups were control (CK, saline), low dose (LS, 1 × 10^6^ CFU mL^−1^), medium dose (MS, 1 × 10^7^ CFU mL^−1^), and high dose (HS, 1 × 10^8^ CFU mL^−1^). Treatments were administered once daily for 14 consecutive days. Body weight was measured at regular intervals throughout the experimental period to monitor growth and physiological responses [17].

#### 2.5.2. Serum Biochemical Assessment

Serum samples were collected and centrifuged at 3000 rpm, 4 °C for 10 min to obtain clear supernatants for biochemical and immunological analyses. The concentrations of high-density lipoprotein cholesterol (HDL-C), low-density lipoprotein cholesterol (LDL-C), and malondialdehyde (MDA) were quantified using enzymatic colorimetric assay kits (Nanjing Jiancheng Bioengineering Institute, Nanjing, China) according to the manufacturer’s instructions. Immunoglobulin G (IgG) and interleukin-2 (IL-2) concentrations in serum were determined by enzyme-linked immunosorbent assay (ELISA) using species-specific kits (Beyotime, Shanghai, China) according to the manufacturers’ protocols.

#### 2.5.3. Histopathological Evaluation

Intestinal samples from mice were fixed in 4% paraformaldehyde solution at pH 7.4, dehydrated through a graded ethanol series, rendered transparent with xylene, and embedded in paraffin. Serial sections of 5 μm thickness were prepared using a rotary microtome (Leica RM2235, Wetzlar, Germany). Sections were stained with hematoxylin and eosin (H&E, Baton Rouge, LA, USA) and mounted using neutral resin. Histopathological alterations were observed under a light microscope (Nikon Eclipse E100, Tokyo, Japan), and images were captured using a digital imaging system (Nikon DS-Ri2, Tokyo, Japan) [18]. To ensure diagnostic accuracy and reduce potential bias, all tissue samples were independently assessed by two board-certified pathologists who were blinded to the experimental groups. Discrepancies between their evaluations were resolved through joint review and consensus.

#### 2.5.4. 16S rDNA Sequencing and Analysis of Intestinal Flora

Genomic DNA was extracted from samples using the cetyltrimethylammonium bromide (CTAB) protocol. Targeting the ribosomal RNA gene of microorganisms, the V3–V4 hypervariable regions of the 16S rRNA gene were amplified by PCR (Eppendorf Mastercycler Nexus, Hamburg, Germany) with universal bacterial primers appended with sample-specific barcodes. Amplification products (470 bp) were confirmed by electrophoresis on a 2% agarose gel (120 V, 30 min). PCR amplicons were then purified with AMPure XT magnetic beads (Beckman Coulter Genomics, Danvers, MA, USA) and precisely quantified using a Qubit 4.0 fluorometer (Invitrogen, Waltham, MA, USA). Upon completion of library preparation, paired-end sequencing (PE250) was performed on an Illumina NovaSeq platform at Beijing Biomake Biotechnology Co., Ltd. (Beijing, China). Raw sequencing reads were filtered using Trimmomatic v0.33 to remove low-quality bases and adapters, after which primer sequences were excised with Cutadapt v1.9.1 to yield high-quality reads. These reads were merged into contiguous sequences via FLASH v1.2.7, and chimeric artifacts were identified and removed using UCHIME v4.2, resulting in the final set of effective sequence data for downstream analysis.

### 2.6. Statistical Analysis

All experimental data were expressed as means ± standard deviation (SD). Statistical analyses and principal component analysis (PCA) were performed using SPSS software version 26.0 (IBM Corp., Armonk, NY, USA). One-way analysis of variance (ANOVA) was employed to determine the significance of differences among groups, and all data were tested for normality using the Shapiro–Wilk test before conducting ANOVA. A *p* < 0.05 was considered statistically significant. PCA was conducted to identify patterns and visualize the relationships among variables across different treatment groups, and the results were presented in terms of principal components that captured the maximum variance within the dataset.

## 3. Results

### 3.1. Isolation and Identification of W. coagulans in Pickle Water

**Figure 1:**
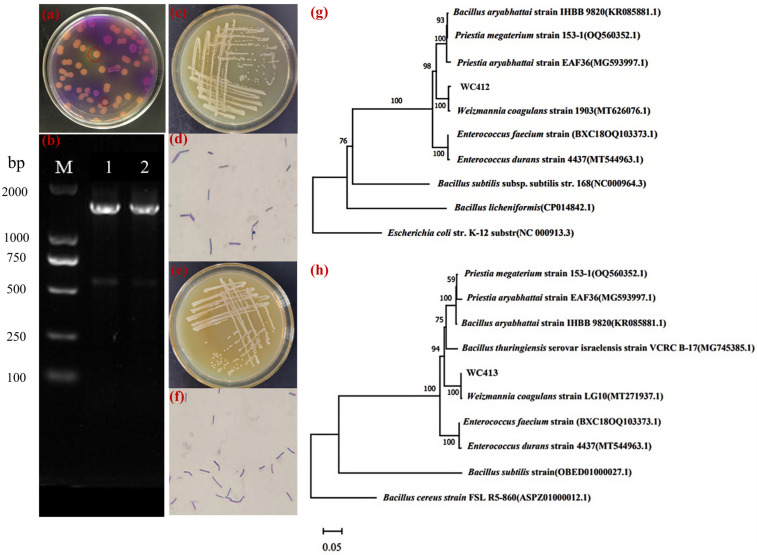
(**a**) Plate screening of colonies from fermented vegetable brine. (**b**) Agarose gel electrophoresis of PCR products; Lane 1 is WC412 and Lane 2 is WC413. (**c**,**d**) Colony morphology and Gram staining (1000×) of strain WC412. (**e**,**f**) Colony morphology and Gram staining (1000×) of strain WC413. (**g**) Phylogenetic tree of strain WC412. (**h**) Phylogenetic tree of strain WC413. Multiple sequence alignment of the core genes from *W. coagulans* strains was performed using MAFFT (version 7.475). Based on the alignment results, a phylogenetic tree was constructed using the Maximum Likelihood method implemented in MEGA software (version 11). Bootstrap support values, calculated from 1000 replicates, are indicated above the branches. The scale bar represents the number of nucleotide substitutions per site.

As shown in Figure 1a, colonies marked with red circles were subjected to streak isolation for purification. Two presumptive *W. coagulans* isolates were preliminarily identified and designated as WC412 and WC413. The two isolates were subsequently cultured on YPD solid medium and incubated at 45 °C for 24 h. Both isolates formed circular colonies with smooth, well-defined margins. The colony surfaces appeared white, opaque, moist, and glossy (Figure 1c,e), exhibiting morphological characteristics consistent with those of *W. coagulans*. Microscopic examination of Gram-stained cells (Figure 1d,f) revealed that both strains were Gram-positive and rod-shaped (staining purple), with no observable endospore structures [19,20].

PCR amplification was carried out using the genomic DNA of each strain as a template, and the resulting products were analyzed by agarose gel electrophoresis. Both WC412 and WC413 displayed distinct bands at approximately 1600 bp (Figure 1b). The amplified 16S rDNA fragments were subsequently sequenced, and the resulting sequences were submitted to the NCBI database for BLAST alignment [21]. The analysis revealed that both sequences shared over 99% similarity with *W. coagulans*. To further verify the taxonomic affiliation, a phylogenetic tree was constructed using MEGA11 software based on highly homologous reference sequences. As shown in Figure 1g,h, strain WC412 clustered closely with *W. coagulans* strain 1903, while WC413 grouped with *W. coagulans* strain LG10, both forming a common clade with high sequence similarity. In conjunction with the results from physiological and biochemical characterization, these findings confirm that strains WC412 and WC413 belong to *W. coagulans*.

### 3.2. Evaluation of Tolerance of W. coagulans

Strains WC412 and WC413, isolated from pickle water, were subjected to tolerance screening alongside laboratory-preserved strains S8, S15, and S17. During large-scale probiotic preparation, the temperature of spray-drying processes typically ranges from 70 to 90 °C. In industrial probiotic feed production, the pelleting temperature generally reaches 80 °C, while aquatic feed processing temperatures can exceed 90 °C [22]. Based on these industrial conditions, the parameters for thermal tolerance assessment in this study were set at 80 to 100 °C for durations of 10 to 20 min. As shown in Table 1, strain S8 exhibited the highest survival rate (82.51%) after heat exposure at 80 °C for 10 min. However, its viability declined significantly (*p* < 0.05) with prolonged heating and increased temperature, and at 90 °C for 10 min, the survival rate dropped to 22.71%. In contrast, strain WC412 maintained a survival rate above 70% after exposure to 80 °C for 10 and 20 min. Under more intense conditions of 90 °C for 20 min and 100 °C for 10 min, WC412 retained survival rates of 51.30% and 34.92%, respectively, both significantly higher than those of the other four strains tested (*p* < 0.05). Collectively, these results indicate that all five tested strains exhibit a certain degree of heat tolerance, with WC412 demonstrating superior thermal resistance.

As the primary organ responsible for food digestion in animals, the stomach plays a pivotal role, with gastric pH serving as a crucial indicator of digestive ecological adaptability. Notably, substantial differences in gastric pH are observed among animals with varying dietary habits—for instance, the abomasum of ruminants maintains a pH of approximately 3.0, whereas in omnivorous species, it may reach as low as 2.0. Furthermore, the retention time of food in the stomach varies depending on its composition [23]. To ensure probiotic efficacy upon reaching the intestine, *W. coagulans* must exhibit robust acid tolerance, enabling it to germinate effectively and exert its beneficial effects. In this study, five candidate strains were subjected to acidic conditions at pH 2.0, 2.5, and 3.0 for 3 h to evaluate their acid resistance. As shown in Table 1, at pH 3.0, all strains except S17 maintained survival rates exceeding 90%. At pH 2.5, strains S8 and WC413 exhibited survival rates above 50%, significantly higher than those of the other strains (*p* < 0.05), while WC412 showed a survival rate of 40.06%. At pH 2.0, only strain S8 retained a survival rate above 10%. Overall, strains S8, WC412, and WC413 demonstrated superior tolerance to an acidic environment.

Bile salts, formed through the conjugation of bile acids with sodium or potassium ions, can compromise cell membrane integrity at high concentrations by altering membrane permeability, ultimately leading to cell death. After passing through the stomach, *W. coagulans* must endure a high bile salt environment for 1–3 h within the intestinal tract; hence, excellent bile salt tolerance is a critical criterion for selecting robust probiotic strains [23,24]. Under 0.1% bile salt conditions, WC413 exhibited the highest survival rate (98.70%), followed by S17 (93.28%) and WC412 (92.21%), while S8 showed the lowest survival rate (48.07%) (Table 1). When the bile salt concentration increased to 0.3%, WC413 (90.93%) and S17 (90.30%) both maintained survival rates above 90%, significantly outperforming the other strains (*p* < 0.05). At a further elevated concentration of 0.5%, WC413 continued to exhibit the highest viability (88.56%, *p* < 0.05), followed by S17 (88.56%), WC412 (78.44%), and S15 (73.29%), with S8 remaining the least tolerant (41.19%). In summary, all strains except S8 demonstrated notable bile salt resistance, with WC413 showing the strongest tolerance, followed by S17, WC412, and S15.

As shown in Table 1, all five *W. coagulans* strains exhibited a certain degree of stress resistance. However, due to the redundancy inherent in multidimensional datasets, direct comparative analysis proves challenging. Therefore, PCA was employed to conduct a comprehensive evaluation. PCA utilizes orthogonal transformation for dimensionality reduction, eliminating inter-variable correlations and extracting core information, thereby enabling an objective and quantitative ranking of multidimensional data [25]. The evaluation of stress tolerance encompassed multiple indicators, including salt resistance, acid–alkali tolerance, and high-temperature survival rates (Table 2).

Following orthogonal transformation, the original variables were reduced to three uncorrelated principal components (Y_1_, Y_2_, and Y_3_), collectively accounting for 94.85% of the total variance, indicating that these three components effectively captured the essential features of stress resistance. Based on the PCA results (Table 2), strain WC412 achieved the highest comprehensive score, significantly surpassing the other four strains, thus demonstrating superior tolerance to combined stress conditions, such as elevated temperature, strong acidity, and high bile salt concentrations. Therefore, WC412 was selected as the target strain for subsequent investigations.

### 3.3. Effect of Supplementation with W. coagulans WC412 on Physiological Metabolism in Mice

**Figure 2:**
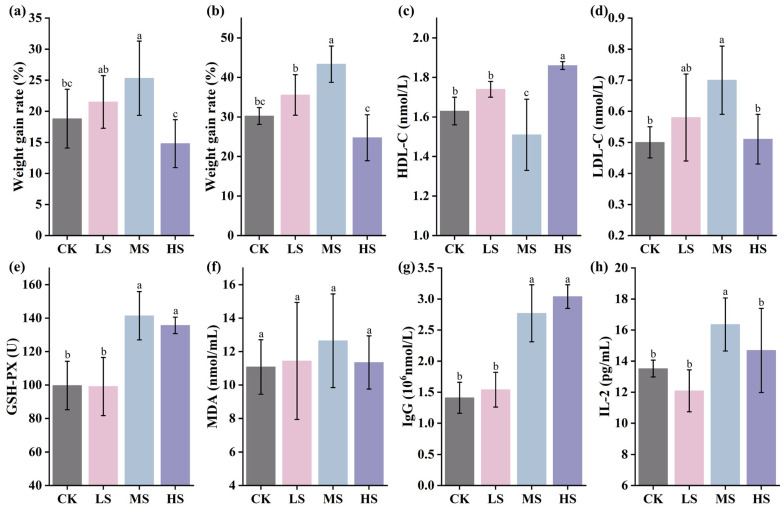
Effects of different doses of WC412 on growth performance and physiological metabolism in mice. (**a**) Body weight changes on day 7 of WC412 administration at varying doses. (**b**) Body weight changes on day 14 of WC412 administration at varying doses. (**c**) High-density lipoprotein cholesterol (HDL-C) levels on day 14 of WC412 administration. (**d**) Low-density lipoprotein cholesterol (LDL-C) levels on day 14 of WC412 administration. (**e**) Glutathione peroxidase (GSH-PX) levels on day 14 of WC412 administration. (**f**) Malondialdehyde (MDA) levels on day 14 of WC412 administration. (**g**) Immunoglobulin G (IgG) levels on day 14 of WC412 administration. (**h**) Interleukin-2 (IL-2) levels on day 14 of WC412 administration. Different letters indicate statistically significant differences between groups (*p* < 0.05).

As shown in Figure 2a,b, during the initial 7-day feeding period, the MS group and LS group exhibited the highest rates of body weight gain, reaching 25.32 ± 5.98% and 21.51 ± 4.24%, respectively (*p* < 0.05). In the later stage of feeding (14 days), the MS group showed the most pronounced body weight gain, achieving 43.36 ± 4.59%, which represents a 13% increase compared with the CK group, indicating a significant growth-promoting effect of WC412 on body weight. Notably, while both the LS and MS groups exhibited positive effects on body weight gain, the HS group showed significantly lower body weight compared to the other groups (*p* < 0.05), suggesting a potential inhibitory effect on growth performance at high dosage levels. To further investigate the effects of WC412 on nutrient metabolism and immune modulation in mice, serum physiological and biochemical parameters were assessed. HDL-C and LDL-C levels serve as key indicators of glucose and lipid metabolic efficiency. As shown in Figure 2c,d, the HDL-C level in the MS group was significantly lower, while the LDL-C level was significantly higher compared to the other groups (*p* < 0.05), suggesting that both the LS and MS groups exhibited improved glucose-lipid metabolism, whereas the HS group presented a distinct metabolic profile. This observation aligns with the body weight data, indicating that WC412 may enhance growth performance by modulating glucose and lipid metabolism. As shown in Figure 2e–h, no significant differences were observed in the levels of MDA, a marker of lipid peroxidation (*p* > 0.05). However, GSH-PX, IgG, and IL-2 levels displayed a consistent trend, with MS and HS groups exhibiting significantly higher levels than the other groups (*p* < 0.05). These findings indicate that WC412 may enhance the antioxidant capacity and immune function of the host organism.

### 3.4. Supplementation of WC412 Regulates the Intestinal Flora of Mice

**Figure 3:**
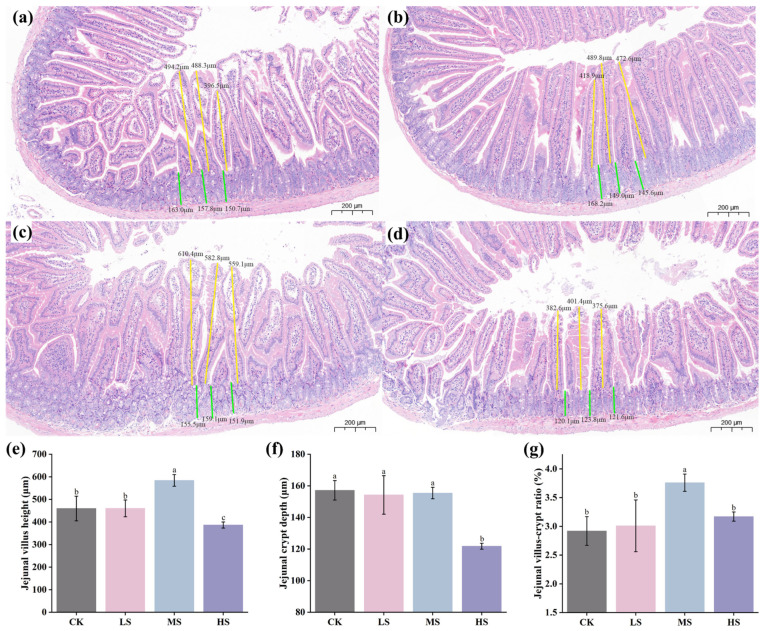
Effects of WC412 administration at different doses on small intestinal morphology. (**a**–**d**) Representative histological sections of the jejunum from the control group (CK), low-dose group (LS), medium-dose group (MS), and high-dose group (HS), respectively. (**e**) Effects of varying doses of WC412 on jejunal villus height. (**f**) Effects of varying doses of WC412 on jejunal crypt depth. (**g**) Effects of varying doses of WC412 on the villus height-to-crypt depth ratio (V/C ratio) in the jejunum. The scale bar represents 200 μm. Different letters indicate statistically significant differences between groups (*p* < 0.05).

As shown in Figure 3a–d, histopathological sections of the jejunum from all four groups exhibited intact mucosal architecture without signs of necrosis or inflammatory infiltration, and the villi were arranged regularly with uniform diameters. Key histological parameters used to assess intestinal structural integrity included villus height (VH), crypt depth (CD), and the villus height-to-crypt depth ratio (VH/CD). The MS group displayed a significantly greater VH compared to the other groups (*p* < 0.05), while the HS group exhibited a markedly reduced CD (*p* < 0.05). Additionally, the VH/CD ratio in the MS group was significantly higher than that in the other groups (*p* < 0.05).

### 3.5. Effects of WC412 on the Gut Microbiota of Mice

#### 3.5.1. Community Diversity Assessment

**Figure 4:**
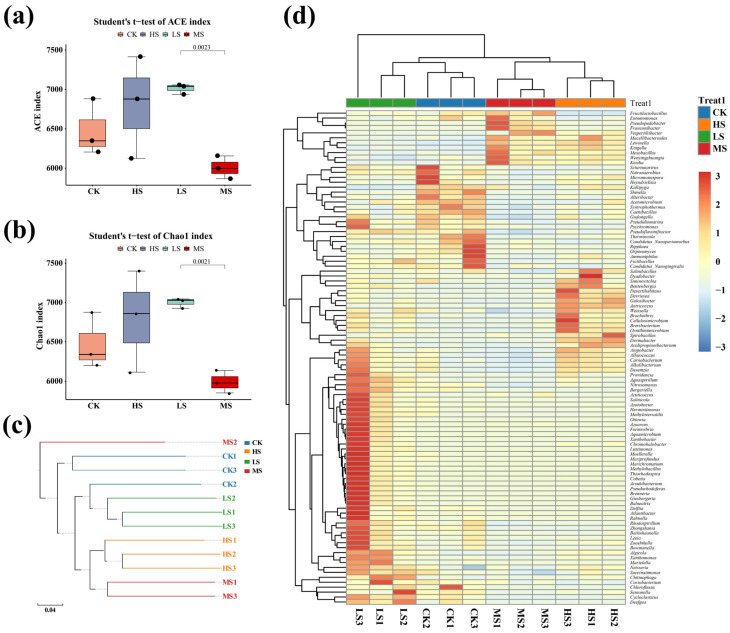
(**a**) α-diversity index based on the ACE metric. (**b**) α-diversity index based on the Chao1 metric. (**c**) UPGMA clustering tree based on species abundance across samples. Different treatment groups are represented by distinct colors, and the branch lengths indicate the degree of similarity in species composition between samples. (**d**) Heatmap of differential genus-level species abundance. The color intensity represents the relative abundance of each microbial genus within the samples, with a gradient ranging from blue (low relative abundance) to red (high relative abundance).

The Abundance-based coverage estimator (ACE) and Chao1 richness estimator (Chao1) indices are commonly used to evaluate microbial richness and community diversity [26]. α-diversity analysis found that the HS group exhibited significantly higher ACE and Chao1 indices compared to the other groups (*p* < 0.05), suggesting enhanced microbial richness and diversity. The diversity indices followed the order: LS > CK > MS. However, no statistically significant differences were observed among these groups (*p* > 0.05) (Figure 4a,b). Overall, mice in the HS group displayed the highest gut microbial diversity, whereas the MS group exhibited the lowest. Hierarchical clustering based on OTU abundance was performed to assess similarities in species composition across samples (Figure 4c). The close clustering of samples within each group indicated a relatively consistent microbiota structure, while clear intergroup differences were observed, reflecting distinct microbial community compositions among the four treatment groups. The relative abundance of microbial genera was quantified at the genus level, and a heatmap was generated to visualize taxa with significant differences (Figure 4d). A total of 164 genera exhibited significant shifts in relative abundance in response to different doses of WC412. Notably, the microbial composition patterns in the LS and CK groups were similar, whereas marked differences were observed between these groups and the HS group. Some genera, such as *Fructilactobacillus*, *Entomomonas*, and *Pseudopedobacter*, showed significant variation in relative abundance across the different treatment groups.

#### 3.5.2. Community Metabolism Pathway Assessment

**Figure 5:**
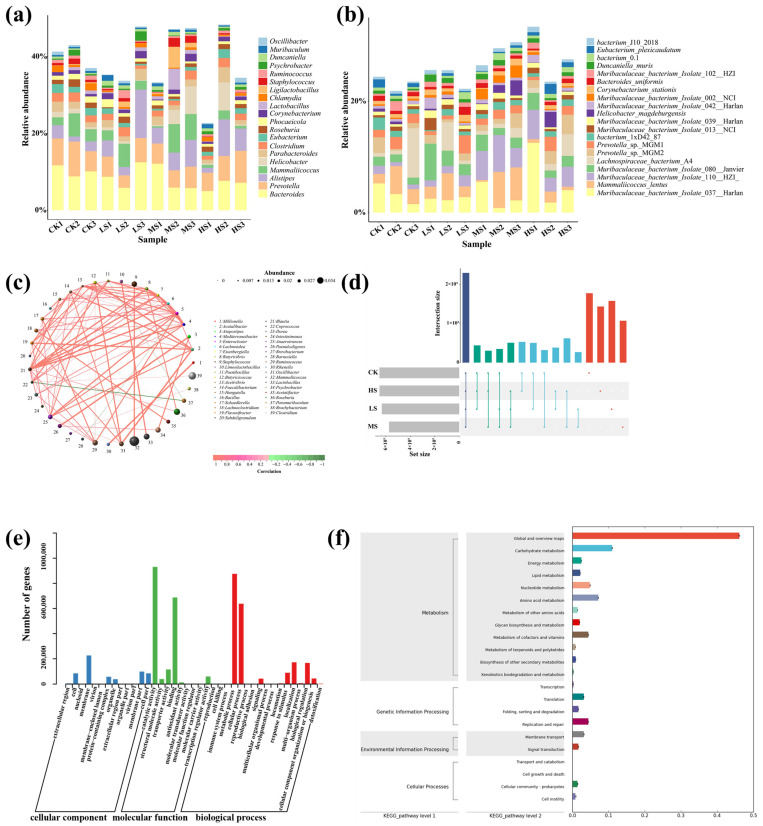
(**a**) Genus-level taxonomic composition bar chart. The bar plot displays the top 20 genera in terms of relative abundance, while all remaining taxa are aggregated and presented as Others. Unassigned refers to taxa lacking taxonomic annotation. (**b**) Species-level taxonomic composition bar chart. The bar plot displays the top 20 species by relative abundance, with other species grouped as Others. Unassigned denotes species without taxonomic classification. (**c**) Microbial correlation network diagram. Circles represent functional genes, with the size of each circle corresponding to its abundance. Lines indicate correlations between genes, line thickness reflects correlation strength, while line color indicates direction-red for positive correlations and green for negative correlations. (**d**) Upset diagram showing differences in the number of detected genes among groups. (**e**) Gene Ontology (GO) functional annotation classification chart. (**f**) KEGG metabolic pathway-related functional gene annotation.

As illustrated in Figure 4d, *W. coagulans* exerted a significant influence on the gut microbial community composition in mice. At the genus level (Figure 5a), unclassified and unassigned taxa constituted more than 50% of the microbial population across all groups, though no clear dose-dependent trend was observed. The intestinal microbiota was predominantly composed of *Bacteroides* and *Prevotella*, jointly accounting for 37.5% of the total community, indicating their dominant status. Notable shifts in genus-level abundance were observed for *Alistipes*, *Mammaliicoccus,* and *Helicobacter* under specific dosage conditions. *Alistipes* exhibited the highest relative abundance in the LS group, whereas *Mammaliicoccus* and *Helicobacter* were markedly enriched in the HS group. In particular, *Helicobacter* abundance surged dramatically in the HS group, with an increase exceeding 249%, suggesting that high-dose treatment may facilitate the proliferation of inflammation-associated bacteria. Similarly, *Ligilactobacillus* showed a striking increase in the HS group, with a relative abundance rise of 1116%. At the species level (Figure 5b), *Muribaculaceae bacterium isolate*, *Mammaliicoccus lentus*, and *Helicobacter magdeburgensis* emerged as dominant taxa. Among them, *Muribaculaceae* displayed a dose-dependent response to WC412, as evidenced by the relative abundance of *Muribaculaceae bacterium isolate* 037, which reached 12.5% in the HS1 group compared to 5.2% in the CK, and *Muribaculaceae bacterium isolate* 110, which showed a 300% increase in the MS and HS groups (5.2%) relative to the CK group (1.5%). The microbial profile of the LS group closely resembled that of the CK group, with distinct differences reflected in an increased abundance of *Turicibacter* (2.91% vs. 1.48% in the CK) and a decreased abundance of *Parabacteroides* by 0.65%. The MS group exhibited the most complex pattern of community perturbation, characterized by a peak in *Alistipes* abundance (7.03%), representing a 187% increase over the CK group (*p* < 0.001), accompanied by a concurrent reduction in the pro-inflammatory genus *Helicobacter* to 0.47%. In contrast, the HS group experienced a notable decline in overall microbial diversity. Within this group, *Ligilactobacillus* reached a relative abundance of 2.31%, showing a dramatic 1116% increase. Conversely, pathogenic genera, such as *Mammaliicoccus* and *Psychrobacter*, showed significant reductions in abundance, decreasing by 66.4% and 62.0%, respectively.

In terms of compositional shifts at the genus and species levels, interdependence or competition among microbial taxa was evident. The microbial correlations were constructed based on the Spearman algorithm (Figure 5c). Among them, *Acetivibrio*, *Faecalibacterium*, *Eisenbergiella*, *Paenibacillus*, and *Enterocloster* were identified as core taxa within the microbial interaction network, each forming either synergistic or antagonistic associations with 12, 8, 7, 7, and 7 other microbial genera, respectively. Notably, most interactions within the network were characterized by mutual dependence. Only a few antagonistic relationships were observed, specifically between *Coprococcus* and *Paramuribaculum*, *Barnesiella* and *Enterocloster*, *Paenibacillus* and *Paramuribaculum*. The competition between probiotic and pathogenic bacteria in the gut, along with microbial metabolic interactions (such as nutrient cross-feeding and antibiotic secretion), can contribute to the emergence of antibiotic resistance genes [3]. Therefore, an analysis of the gene composition was conducted to infer the metabolic functions of the gut microbiota. As shown in Figure 5d, the CK group exhibited the highest number of gene fragments, whereas the MS group displayed the fewest. Moreover, the CK and HS groups showed the greatest similarity in their gene profiles. Functional annotation of the detected genes revealed that most were involved in cellular component, molecular function, and biological process categories. Specifically, catalytic activity and binding were the dominant terms under molecular function, while the metabolic process and reproductive process represented the primary biological processes (Figure 5e). Further KEGG pathway enrichment analysis indicated that microbial life activities were predominantly associated with Metabolism, Genetic Information Processing, Environmental Information Processing, and Cellular Processes (Figure 5f).

## 4. Discussion

The survival rate of probiotic strains during industrial processing and following ingestion into the host organism is a critical prerequisite for their effective probiotic functionality. Adverse conditions, including high-temperature treatments in industrial settings and the presence of strong gastric acid and bile salts in the gastrointestinal tract, significantly impact probiotic viability [27]. In this context, the stress tolerance of probiotic candidates becomes a fundamental criterion for selection. In the present study, all five *W. coagulans* strains exhibited a certain degree of adaptability under extreme conditions. This can be attributed to the fact that *W. coagulans* is a Gram-positive, facultatively anaerobic, spore-forming probiotic bacterium. Its facultative anaerobic nature and ability to produce endospores confer strong viability during drying processes, while its spores are capable of rapid germination in culture media [27]. Beyond survival, functional efficacy in host systems is a pivotal measure of probiotic performance. Probiotics have been widely applied in livestock and poultry farming, primarily through the modulation of intestinal microecological balance in the host, thereby influencing overall health, growth performance, and disease resistance [27]. Among measurable outcomes, body weight gain serves as a practical and direct indicator of animal production efficiency. The body weight results indicated that the LS and MS groups enhanced the growth performance of mice (Figure 2), which holds potential value for applications in the livestock and poultry farming industry. *W. coagulans*, a close relative of *Bacillus coagulans*, has been shown to colonize the gut and secrete hydrolytic enzymes, such as amylases and proteases, which facilitate the breakdown of complex nutrients, enhancing their bioavailability and contributing to improved body weight gain in poultry [26]. This aligns with our findings, wherein the MS group supplemented with WC412 showed a significant increase in body weight compared to the control group, indicating that optimized microbial supplementation offers a promising route for improving livestock productivity. Meanwhile, histopathological analysis of intestinal structure indicated that WC412 contributed to the maintenance of intestinal integrity, with the MS group exhibiting the most favorable ratio of jejunal villus height to crypt depth (Figure 3). Intestinal morphological integrity is a fundamental physiological basis for maintaining gut health, directly influencing nutrient absorption efficiency and immune homeostasis [28]. The ratio of villus height to crypt depth serves as a critical indicator for assessing the efficiency of nutrient absorption, with a higher ratio being positively correlated with enhanced absorptive capacity. Moreover, changes in crypt depth are closely associated with the mitotic proliferation of intestinal villus epithelial cells [25]. Wang et al. [28] reported that *W. coagulans* enhanced intestinal function by promoting epithelial cell maturation, thereby increasing villus length. Collectively, these results indicate that moderate supplementation with WC412 (MS) effectively preserves intestinal morphological integrity by optimizing the villus–crypt architecture, a finding consistent with observed improvements in body weight gain and serum HDL-C/LDL-C levels, indicative of enhanced glyco-lipid metabolic performance.

In addition to growth promotion, probiotic supplementation can modulate immune responses through both direct and indirect mechanisms. Previous studies have confirmed that probiotics can enhance immune function by activating the innate immune system and modulating inflammatory responses, such as through the regulation of antioxidant enzymes [29]. In addition, probiotics exert immunoregulatory effects by modulating the expression of pro-inflammatory and anti-inflammatory cytokines, which, in turn, respond to microbial antigens to activate innate immune pathways [26]. This study found that WC412 supplementation elevated levels of GSH-PX, IgG, and IL-2, particularly in the medium-dose group, indicating its immunostimulatory potential (Figure 2). These immunological benefits are often mediated by the probiotic’s influence on the gut microbiota—a complex ecosystem increasingly recognized as a key player in host immune regulation. They can directly enhance the activity of immune cells and indirectly regulate immune responses by optimizing the gut microbiota composition. Although the causal relationship between pathological states and gut microbiota remains a subject of debate, substantial evidence indicates that a healthy microbial community is a critical component in establishing a long-term defense system against pathogenic invasion [30]. Therefore, evaluating the gut microbial community offers a valuable approach to further elucidate the potential mechanisms by which *W. coagulans* influences the growth performance of economically important animals.

Gut microbiota dysbiosis typically exerts profound and deleterious effects on the productivity and overall health status of economically important animals, making the restoration of intestinal microbial homeostasis critical for optimizing production outcomes [7]. In this context, we investigated whether WC412 could mitigate the adverse effects on gut microbiota while improving growth performance and immune parameters in mice. As anticipated, we observed a general increase in microbial diversity indices in mice treated with LS and MS of WC412, indicating enhanced microbial richness and evenness (Figure 4). Firstly, the LS and CK groups exhibited limited differences in microbial genera, with the exception of *Brevibacterium*. The most notable changes involved an increase in probiotic genera, such as *Carnobacterium* and *Weissella*, which are known to inhibit the proliferation of pathogenic bacteria, such as *Salmonella*, and enhance intestinal barrier function [21]. However, an elevated abundance of the enteropathogenic genus *Providencia*, which is associated with intestinal mucosal damage, was also found previously [31]. This suggests that the modulatory effect of *W. coagulans* may exhibit bidirectional characteristics. Although the microbial diversity in the MS group was lower than that observed in the LS group, the fold change in specific microbial taxa increased significantly. Notably, the proliferation of beneficial acid-producing genera, such as *Limosilactobacillus*, *Pontibacter*, and *Fructilactobacillus*, was observed. These probiotics are known to produce lactic acid, acetic acid, and short-chain fatty acids (SCFAs), which inhibit pathogenic species, such as *Salmonella* and *Clostridium perfringens*, thereby enhancing intestinal barrier integrity [21]. Moreover, previous clinical studies conducted in healthy adults have shown that an intervention with *Bacillus coagulans* SANK70258 significantly increased the abundance of *Bifidobacterium* in the gut, while concurrently reducing fecal pH, ammonia concentration, and the levels of harmful metabolites, such as p-cresol and indole [21]. These findings are consistent with the results observed in our study. Simultaneously, WC412 was capable of positively regulating the suppression of pathogenic bacterial enrichment, including *Providencia*, *Trueperella*, and *Megasphaera*, all of which are associated with inflammatory responses that impair animal growth performance [31,32]. However, it is noteworthy that MS treatment also promoted the proliferation of the opportunistic pathogen *Proteus* (fold change 1.5), which is known to cause urinary tract infections and septicemia, highlighting a potential risk associated with microbial imbalance.

HS administration resulted in a marked decline in the gut microbial diversity indices of mice, indicating a significant disruption in both microbial richness and evenness. Notably, this disruption was characterized by the suppression of several potentially beneficial genera, including *Roseburia*, *Coprococcus*, *Prevotella*, and members of the *Lachnospiraceae* family, alongside an increase in potentially harmful or opportunistic pathogens, such as *Providencia*, *Citrobacter*, *Actinobacillus*, *Trueperella*, and *Neisseria*. Among these, *Roseburia* is widely recognized as a beneficial commensal bacterium that plays a crucial role in maintaining intestinal barrier integrity, exerting anti-inflammatory effects, and promoting mucin secretion. Similarly, *Coprococcus*, *Prevotella*, and *Lachnospiraceae* are functionally important for carbohydrate and fiber metabolism and regulation of gut motility [33]. In contrast, *Providencia* and *Neisseria* are opportunistic pathogens known to cause septicemia and diarrhea in livestock species [32]. Citrobacter, *Actinobacillus*, and *Trueperella* are pro-inflammatory taxa associated with pathological damage to the gastrointestinal, respiratory, and reproductive systems, with some capable of producing endotoxins [21]. Of particular concern was the significant enrichment of environmental microbes, such as *Pseudoxanthomonas*, *Williamsia*, and *Kutzneria* within the intestinal tract [34]. We speculate that these bacteria may competitively deplete essential nutrients, thereby impairing host nutrient absorption and reducing growth performance in economically important animals. These microbial shifts align with observed declines in overall species richness, culturable microbial abundance, and growth performance metrics in the HS group, as well as with immunological impairments, collectively indicating a negative impact on host health.

Microorganisms are generally recognized as key contributors to food digestion, primarily due to their ability to secrete a wide array of hydrolytic enzymes, including carbohydrases, proteases, lipases, nucleases, phosphatases, and other hydrolases [21]. Differential gene expression analysis revealed that microbial metabolic processes were mainly enriched in the pathways related to carbohydrate metabolism, amino acid metabolism, metabolism of cofactors and vitamins, energy metabolism, lipid metabolism, and nucleotide metabolism (Figure 5f). The intestinal microbiota exerts a profound influence on the growth performance of economically important animals through a complex carbohydrate metabolic network. The core mechanism lies in the fermentation of indigestible dietary polysaccharides (e.g., cellulose, hemicellulose, and resistant starch) and host-derived mucin oligosaccharides, resulting in the production of SCFAs (primarily acetate, propionate, and butyrate), organic acids, and metabolic energy [35]. On one hand, SCFAs—particularly butyrate—serve as the preferred energy substrate for intestinal epithelial cells, stimulating crypt cell proliferation and villus morphological development, thereby enhancing intestinal barrier function and tight junction integrity [28]. This contributes to an increased surface area and efficiency for nutrient absorption, while also promoting the secretion of gastrointestinal peptides, such as GLP-2, which improves local blood flow [36]. On the other hand, SCFAs, like propionate, can enter systemic circulation via the portal vein, where they serve as gluconeogenic precursors or participate directly in energy metabolism. This reduces the host’s demand for glycolysis and lipolysis, conserving energy for protein deposition. Additionally, SCFAs modulate host metabolic pathways by activating G-protein–coupled receptors (e.g., GPR41/43) and inhibiting histone deacetylases (HDACs), thereby regulating energy homeostasis, insulin sensitivity, and the expression of the genes involved in lipogenesis [21,37,38]. Moreover, microbial degradation of anti-nutritional factors, such as xylan, reduces digesta viscosity, improving nutrient–enzyme interaction and digestive efficiency. Certain symbiotic microbial populations also competitively inhibit the proliferation of pathogenic species, such as *Clostridium perfringens*, thereby alleviating inflammatory responses and reducing energy expenditure associated with mucosal immune activation. Meanwhile, some genera, such as *Coprococcus*, *Prevotella*, *Lachnospiraceae*, *Carnobacterium*, *Pontibacter*, and *Fructilactobacillus*, were significantly enriched (Figure 4d). These microbial taxa are widely recognized for their involvement in carbohydrate and energy metabolism. Taken together, the efficiency of microbial carbohydrate metabolism constitutes a central biological determinant of nutrient utilization and growth performance in animals.

KEGG pathway enrichment analysis revealed that WC412 significantly promoted fatty acid metabolism within the gut microbiota (Figure 5f). Fatty acids and SCFAs are essential for maintaining intestinal microbial homeostasis and overall gut health [39]. SCFAs are known to regulate tight junction integrity, intestinal permeability, and the expression of immune cytokines. As primary metabolic end-products of gut microbiota, fatty acids are closely associated with intestinal physiological functions [28]. Simultaneously, the proliferation of potentially beneficial gut microorganisms, including various strains of *Limosilactobacillus*, *Pontibacter*, and *Fructilactobacillus*, was observed (Supplementary Materials). These bacteria are widely recognized for their capacity to enhance SCFA production. Notably, *W. coagulans* itself serves as a major source of SCFAs, including lactate, acetate, and butyrate [20]. The SCFAs secreted by this strain may act by sustaining the energy supply to epithelial cells (specifically intestinal mucosal cells), upregulating the expression and assembly of tight junction proteins, such as occludin and claudin, thereby reinforcing intercellular junctions, and reducing intestinal permeability [38,39,40]. It was reported that *W. coagulans* improved lipid distribution in obese mice and prevented high-fat diet-induced hepatic steatosis. This effect was mediated through the regulation of lipid metabolism, which led to a reduction in pro-inflammatory cytokine levels and subsequently alleviated hepatic fat accumulation [41]. These findings are consistent with observed improvements in immunological markers and histopathological features (Figure 2 and Figure 3), indicating that WC412 may enhance intestinal barrier function and integrity. Based on these observations, we propose that WC412 improves intestinal physiological characteristics by modulating gut microbial structure and increasing the abundance of SCFA-producing bacteria. It is noteworthy that amino acid metabolism by the gut microbiota exhibits a dual nature characterized by “competition–compensation” dynamics. Microbial degradation of dietary proteins and endogenous mucin glycoproteins leads to the release of free amino acids, which facilitates rapid absorption of nutrients by the small intestinal mucosa. The gut microbiota is also capable of synthesizing certain amino acids, such as lysine and threonine, as well as bioactive derivatives, like indolepropionic acid and kynurenine, which directly contribute to host protein synthesis or function as signaling molecules [42]. Among these, indole derivatives enhance intestinal barrier function by activating the aryl hydrocarbon receptor (AhR) and pregnane X receptor (PXR), while simultaneously attenuating inflammation through inhibition of the NF-κB pathway, thereby reducing the amino acid demand associated with mucosal repair [42]. However, microorganisms can also competitively consume essential amino acids, such as branched-chain amino acids, thereby diminishing the host’s bioavailable amino acid pool. In addition, under conditions of increased nitrogen load, certain gas-producing microbes may generate excessive ammonia, leading to intestinal bloating, epithelial damage, and an elevated hepatic detoxification burden [43]. Unfortunately, *Proteus*, a genus implicated in the regulation of ammonia metabolism, was found to be significantly upregulated in the LS group.

For microorganisms, nucleotide metabolism is an essential pathway for the replication and transcription of genetic material during their proliferation. Additionally, microbial nucleotide metabolism serves as an endogenous salvage pathway, wherein gut microbiota utilizes dietary nucleic acids or nucleoside/nucleotide precursors released from apoptotic host cells (e.g., 5′-phosphoribose and glutamine) to synthesize purines (adenosine and guanosine) and pyrimidines (uridine and cytidine) via de novo or salvage pathways [44]. A portion of these nucleotides can be absorbed and utilized by host intestinal epithelial cells. As critical substrates for cellular proliferation, these nucleotides directly contribute to the division of intestinal crypt stem cells, regeneration of villi, and differentiation of immune cells, such as lymphocytes [35]. Overall, microbial metabolic activity supports the maintenance of intestinal homeostasis and immune balance, thereby reducing maintenance energy requirements and enabling the effective reallocation of resources toward growth. It is important to note, however, that these beneficial effects are primarily attributed to probiotic microorganisms. While this study has demonstrated the promising probiotic potential of *W. coagulans* WC412, several limitations warrant consideration. The murine model employed here provides foundational insights into WC412′s probiotic functions but may not fully recapitulate the complex gastrointestinal physiology, dietary patterns, or microbial ecosystems of livestock species (e.g., poultry, swine, or ruminants). The 14-day supplementation period assessed acute physiological responses but cannot elucidate long-term effects on host health, microbial stability, or potential adaptive resistance mechanisms. Extended trials monitoring growth performance, immune parameters, and microbiota dynamics over production cycles are needed. These limitations highlight critical avenues for future research to advance WC412 toward practical applications in animal production.

## 5. Conclusions

Two novel strains of *W. coagulans* (WC412 and WC413) were isolated from fermented kimchi brine. Among them, WC412 exhibited superior resistance to heat, acid, and bile stress. In vivo supplementation with WC412 significantly enhanced body weight gain, improved lipid metabolism, boosted antioxidant and immune responses, and favorably modulated gut microbiota composition in mice. Medium-dose treatment achieved optimal outcomes, while high-dose administration led to potential microbial imbalance. These findings highlight WC412 as a promising probiotic candidate for improving growth performance and gut health in animal production.

## Figures and Tables

**Table 1:** Assessment of the tolerance of *W. coagulans* to high temperatures, acids, and bile salts.

Coercive Conditions			Survival Rate (%)				
			S8	S15	S17	WC412	WC413
Tolerance to high temperatures	80 °C	10 min	82.51 ± 3.42 ^Aa^	69.95 ± 0.86 ^Ab^	55.89 ± 0.39 ^Ac^	71.22 ± 2.58 ^Ab^	55.30 ± 1.52 ^Ac^
		20 min	53.60 ± 1.91 ^Bab^	56.68 ± 0.87 ^Bb^	51.07 ± 0.87 ^Bc^	73.31 ± 0.67 ^Aa^	57.06 ± 1.51 ^Ab^
	90 °C	10 min	22.71 ± 1.32 ^Cc^	19.83 ± 1.76 ^Cc^	37.95 ± 0.56 ^Cb^	59.65 ± 1.99 ^Ba^	43.11 ± 1.31 ^Bb^
		20 min	17.73 ± 1.16 ^Cd^	9.49 ± 0.61 ^De^	24.82 ± 1.15 ^Dc^	51.35 ± 0.70 ^Ca^	44.86 ± 2.96 ^Bb^
	100 °C	10 min	16.06 ± 0.11 ^Cd^	3.03 ± 0.35 ^Ee^	21.28 ± 1.17 ^Ec^	34.92 ± 1.51 ^Da^	28.71 ± 0.47 ^Cb^
		20 min	19.61 ± 0.87 ^Ca^	1.36 ± 0.06 ^Ec^	7.62 ± 0.50 ^Fb^	1.24 ± 0.03 ^Ec^	2.91 ± 0.07 ^Dc^
Tolerance to acids	pH 2		15.17 ± 0.61 ^Ca^	9.04 ^Cb^	4.36 ± 0.62 ^Cc^	3.17 ± 0.11 ^Cc^	3.90 ± 0.34 ^Cc^
	pH 2.5		55.05 ± 0.31 ^Ba^	12.06 ± 0.14 ^Bd^	31.20 ± 2.04 ^Bc^	40.06 ± 1.82 ^Bb^	50.22 ± 2.20 ^Ba^
	pH 3		96.05 ± 1.23 ^Aa^	90.12 ± 4.11 ^Aa^	69.50 ± 1.89 ^Ab^	95.07 ± 0.03 ^Aa^	97.35 ± 1.07 ^Aa^
Tolerance to bile salts	0.10%		48.07 ± 0.48 ^Ad^	85.67 ± 1.09 ^Ac^	93.28 ± 2.24 ^Ab^	92.21 ± 0.96 ^Ab^	98.70 ± 0.01 ^Aa^
	0.30%		45.34 ± 1.19 ^Bc^	82.07 ± 1.54 ^Ab^	90.30 ± 5.22 ^Aa^	81.55 ± 1.86 ^Bb^	90.93 ± 0.37 ^Ba^
	0.50%		41.19 ± 0.45 ^Cd^	73.29 ± 3.40 ^Bc^	85.82 ± 0.75 ^Aab^	78.44 ± 0.92 ^Bbc^	88.56 ± 0.57 ^Ca^

Note: Different letters indicate statistically significant differences between groups (*p* < 0.05).

**Table 2:** PCA-based principal component scores and composite scores for five strains of bacteria.

Ingredient	Initial Eigenvalue			Rotational Load Sum of Squares		
	Total	Percentage of Variance (%)	Cumulative (%)	Total	Percentage of Variance (%)	Cumulative (%)
1	6.733	56.108	56.108	6.102	50.85	50.85
2	3.25	27.086	83.195	3.331	27.759	78.609
3	1.399	11.66	94.854	1.949	16.246	94.854
**Serial No.**	**Strain**	**Y_1_ Score**	**Y_2_ Score**	**Y_3_ Score**	**Combined Score**	**Ranking**
1	WC412	1.97	1.96	0.95	1.75	1
2	WC413	1.95	0.63	−0.64	1.19	2
3	S17	1.05	−1.69	−1.34	−0.02	3
4	S15	−0.8	−2.11	1.5	−0.85	4
5	S8	−4.18	1.2	−0.47	−2.07	5

## Data Availability

The raw data supporting the conclusions of this article will be made available by the authors on request.

## Supplementary Materials

The following supporting information can be downloaded at: https://www.mdpi.com/article/10.3390/ani15162446/s1.

## References

[1] Barreto-Cruz O.T., Henao Zambrano J.C., Ospina Barrero M.A., Castañeda-Serrano R.D. (2024). Effects of *Tithonia diversifolia* extract as a feed additive on digestibility and performance of hair lambs. Animals.

[2] Hoseinifar S.H., Faheem M., Liaqat I., Van Doan H., Ghosh K., Ringø E. (2024). Promising probiotic candidates for sustainable aquaculture: An updated review. Animals.

[3] Zamojska D., Nowak A., Nowak I., Macierzyńska-Piotrowska E. (2021). Probiotics and postbiotics as substitutes of antibiotics in farm animals: A Review. Animals.

[4] Zhou Y., Zeng Z., Xu Y., Ying J., Wang B., Majeed M., Majeed S., Pande A., Li W. (2020). Application of *Bacillus coagulans* in animal husbandry and its underlying mechanisms. Animals.

[5] Muhammad Z., Anjum M.Z., Akhter S., Irfan M., Amin S., Jamal Y., Khalid S., Ghazanfar S. (2024). Effect of Lactobacillus plantarum and *Pediococcus pentosaceus* on the growth performance and morphometry of the genetically improved farmed tilapia (*Oreochromis niloticus*). Pak. J. Zool..

[6] Slizewska K., Markowiak-Kopec P., Zbikowski A., Szeleszczuk P. (2020). The effect of synbiotic preparations on the intestinal microbiota and her metabolism in broiler chickens. Sci. Rep..

[7] Kober A.K.M.H., Riaz Rajoka M.S., Mehwish H.M., Villena J., Kitazawa H. (2022). Immunomodulation potential of probiotics: A novel strategy for improving livestock health, immunity, and productivity. Microorganisms.

[8] Mingmongkolchai S., Panbangred W. (2018). Bacillus probiotics: An alternative to antibiotics for livestock production. J. Appl. Microbiol..

[9] Hou J., Lian L., Lu L., Gu T., Zeng T., Chen L., Xu W., Li G., Wu H., Tian Y. (2023). Effects of dietary *Bacillus coagulans* and Tributyrin on growth performance, serum antioxidants, intestinal morphology, and cecal microbiota of growing yellow-feathered broilers. Animals.

[10] Chang X., Chen Y., Feng J., Huang M., Zhang J. (2021). Amelioration of Cd-induced bioaccumulation, oxidative stress and immune damage by probiotic *Bacillus coagulans* in common carp (*Cyprinus carpio* L.). Aquac. Rep..

[11] Yu Y., Wang C., Wang A., Yang W., Lv F., Liu F., Liu B., Sun C. (2018). Effects of various feeding patterns of *Bacillus coagulans* on growth performance, antioxidant response and Nrf2-Keap1 signaling pathway in juvenile gibel carp (*Carassius auratus gibelio*). Fish Shellfish Immunol..

[12] Yi L., Qi T., Hong Y., Deng L., Zeng K. (2020). Screening of bacteriocin-producing lactic acid bacteria in Chinese homemade pickle and dry-cured meat, and bacteriocin identification by genome sequencing. LWT.

[13] Madushanka D., Vidanarachchi J.K., Kodithuwakku S., Nayanajith G.R.A., Jayatilake S., Priyashantha H. (2025). Isolation and characterization of probiotic lactic acid bacteria from fermented traditional rice for potential applications in food and livestock production. Appl. Food Res..

[14] Xu Y., Xiong T., Zhang L., Du T., Madjirebaye P., Zhao M., Kang X. (2025). Novel lactic acid bacteria with anti-hyperglycaemic properties: In vitro screening and probiotic assessment. Food Biosci..

[15] Banik A., Anjum H., Habib H., Abony M., Begum A., Ahmed Z. (2023). Characterization of lactic acid bacteria isolated from street pickles of Dhaka, Bangladesh. Heliyon.

[16] Sadeghi M., Panahi B., Mazlumi A., Hejazi M.A., Komi D.E.A., Nami Y. (2022). Screening of potential probiotic lactic acid bacteria with antimicrobial properties and selection of superior bacteria for application as biocontrol using machine learning models. LWT.

[17] Yang X., Ren R., Lang X., Li X., Qin L., Zeng H. (2024). Effect of whole-grain Tartary buckwheat fermentation with *Monascus* purpureus on the metabolic syndrome and intestinal flora in mice. Food Biosci..

[18] Jentzer A., Fauteux-Daniel S., Verhoeven P., Cantais A., Novoa M.Y., Jospin F., Chanut B., Rochereau N., Bourlet T., Roblin X. (2022). Impact of dextran-sodium-sulfate-induced enteritis on murine cytomegalovirus reactivation. Viruses.

[19] Lertsriwong S., Glinwong C. (2020). Newly-isolated hydrogen-producing bacteria and biohydrogen production by *Bacillus coagulans* MO11 and *Clostridium beijerinckii* CN on molasses and agricultural wastewater. Int. J. Hydrogen Energy.

[20] Mai X., Huang F., Feng W., Zhang X., Li J., Huang Y., And Zeng X. (2025). *Weizmannia coagulans*: Screening, functional properties, and current applications in the food industry. Food Rev. Int..

[21] Zhang S., Zhang D., Wang T., Lee S., Lim C., Zhao Y., Li P. (2023). In vitro and in vivo evaluation of efficacy and safety of *Weizmannia coagulans* HOM5301 for boosting immunity. J. Funct. Foods.

[22] Huang S., Vignolles M.L., Chen X.D., Le Loir Y., Jan G., Schuck P., Jeantet R. (2017). Spray drying of probiotics and other food-grade bacteria: A review. Trends Food Sci. Technol..

[23] Konuray Altun G., Erginkaya Z. (2021). Identification and characterization of *Bacillus coagulans* strains for probiotic activity and safety. LWT.

[24] Sreenadh M., Kumar K.R., Nath S. (2022). In vitro evaluation of *Weizmannia coagulans* strain LMG S-31876 isolated from fermented rice for potential probiotic properties, safety assessment and technological properties. Life.

[25] Yang Z.L., Shi Y.Q., Li P.L., Pan K.H., Li G.Q., Li X.G., Yao S., Zhang D.H. (2022). Application of principal component analysis (PCA) to the evaluation and screening of multiactivity fungi. J. Ocean Univ..

[26] Fu R., Chen D., Tian G., Zheng P., Mao X., Yu J., He J., Huang Z., Luo Y., Yu B. (2019). Effect of dietary supplementation of *Bacillus coagulans* or yeast hydrolysates on growth performance, antioxidant activity, cytokines and intestinal microflora of growing-finishing pigs. Anim. Nutr..

[27] Wang L., Wang J., Du L., Fang X., Liao Z. (2022). Application of *Weizmannia coagulans* in the medical and livestock industry. Ann. Microbiol..

[28] Wang Z.B., Guo Z.T., Liu L.B., Ren D.X., Zu H., Li B.L., Liu F. (2024). Potential probiotic *Weizmannia coagulans* WC10 improved antibiotic-associated diarrhea in mice by regulating the gut microbiota and metabolic homeostasis. Probiotics Antimicrob. Proteins.

[29] Yin G., Li W., Lin Q., Lin X., Lin J., Zhu Q., Jiang H., Huang Z. (2014). Dietary administration of laminarin improves the growth performance and immune responses in *Epinephelus coioides*. Fish Shellfish Immunol..

[30] Plaza-Diaz J., Ruiz-Ojeda F.J., Gil-Campos M., Gil A. (2019). Mechanisms of action of probiotics. Adv. Nutr..

[31] Malviya M., Kale-Pradhan P., Coyle M., Giuliano C., Johnson L.B. (2024). Clinical and drug resistance characteristics of providencia infections. Microorganisms.

[32] Galán-Relaño A., Gómez-Gascón L., Barrero-Domínguez B., Luque I., Jurado-Martos F., Vela A.I., Sanz-Tejero C., Tarradas C. (2020). Antimicrobial susceptibility of Trueperella pyogenes isolated from food-producing ruminants. Vet. Microbiol..

[33] Faden H. (2022). The role of Faecalibacterium, Roseburia, and Butyrate in inflammatory bowel disease. Dig. Dis..

[34] Ma M.X., Ye X.Y., Zhang S., Wang X.L., Qiu X.M. (2022). Analysis of intestinal flora and environmental microbial diversity of Takifugu rubripes. Isr. J. Aquac.-Bamidgeh.

[35] Burcelin R., Luche E., Serino M., Chabo C. (2009). The intestinal flora: New concepts for the regulation of energetic metabolism. Sang Thrombose Vaisseaux.

[36] Li D.Y., Gao Y.H., Cui L.R., Li Y., Ling H., Tan X., Xu H.Y. (2023). Integrative analysis revealed the role of glucagon-like peptide-2 in improving experimental colitis in mice by inhibiting inflammatory pathways, regulating glucose metabolism, and modulating gut microbiota. Front. Microbiol..

[37] Sun P., Liu J.C., Chen G.N., Guo Y.L. (2025). The role of G protein-coupled receptors in the regulation of orthopaedic diseases by gut microbiota. Nutrients.

[38] Zhang S.Q., Hou R., Wang Y.C., Huang Q.Y., Lin L., Li H.X., Liu S., Jiang Z.J., Huang X.P., Xu X.R. (2024). Xenobiotic metabolism activity of gut microbiota from six marine species: Combined taxonomic, metagenomic, and in vitro transformation analysis. J. Hazard Mater..

[39] Yan X.X., Li J.N., Wu D. (2023). The role of short-chain fatty acids in acute pancreatitis. Molecules.

[40] Zhang S., Li P., Lee S., Wang Y., Tan C., Shang N. (2024). *Weizmannia coagulans*: An ideal probiotic for gut health. Food Sci. Hum. Wellness.

[41] Urtasun R., Díaz-Gómez J., Araña M., Pajares M.J., Oneca M., Torre P., Jiménez M., Munilla G., Barajas M., Encío I. (2020). A combination of apple vinegar drink with *Bacillus coagulans* ameliorates high fat diet-induced body weight gain, insulin resistance and hepatic steatosis. Nutrients.

[42] Zhang Y.P., Sun M.J., Liu Y.C., Chu T., Liu X.J., Cui Z.H., Jin S.Z., Yuan X.C. (2023). Gut microbiota adaptation to low and high carbohydrate-to-protein ratio diets in grass carp (*Ctenopharyngodon idella*). Aquac. Rep..

[43] Li Y.T., Zhang R.X., Li X., Li J.H., Ji W.B., Zeng X.Y., Bao J. (2021). Exposure to the environmental pollutant ammonia causes changes in gut microbiota and inflammatory markers in fattening pigs. Ecotoxicol. Environ. Saf..

[44] Wang X.Y., Zhang K., Zhang J.Y., Xu G.W., Guo Z.T., Lu X.R., Liang C.H., Gu X.Y., Huang L.P., Liu S.Q. (2025). Cordyceps militaris solid medium extract alleviates lipopolysaccharide-induced acute lung injury via regulating gut microbiota and metabolism. Front. Immunol..

